# Sputtering-Deposited Ultra-Thin Ag–Cu Films on Non-Woven Fabrics for Face Masks with Antimicrobial Function and Breath NO_x_ Response

**DOI:** 10.3390/ma17071574

**Published:** 2024-03-29

**Authors:** Xuemei Huang, Qiao Hu, Jia Li, Wenqing Yao, Chun Wang, Yun Feng, Weijie Song

**Affiliations:** 1 Ningbo Institute of Materials Technology and Engineering, Chinese Academy of Sciences, Ningbo 315201, China; huangxuemei@nimte.ac.cn (X.H.); huqiao9419@163.com (Q.H.); 2 University of Chinese Academy of Sciences, Beijing 100049, China; 3 Department of Chemistry, Tsinghua University, Beijing 100084, China; yaowq@tsinghua.edu.cn; 4 Ningbo Customs Technology Center, Ningbo 315012, China; 13616785050@163.com (C.W.); 13645749255@163.com (Y.F.)

**Keywords:** ultra-thin Ag–Cu film, antimicrobial fabric, face masks, breath NO_x_

## Abstract

The multifunctional development in the field of face masks and the growing demand for scalable manufacturing have become increasingly prominent. In this study, we utilized high-vacuum magnetron sputtering technology to deposit a 5 nm ultra-thin Ag–Cu film on non-woven fabric and fabricated ultra-thin Ag–Cu film face masks. The antibacterial rates against *Escherichia coli* and *Staphylococcus aureus* were 99.996% and 99.978%, respectively, while the antiviral activity against influenza A virus H1N1 was 99.02%. Furthermore, the mask’s ability to monitor respiratory system diseases was achieved through color change (from brownish-yellow to grey-white). The low cost and scalability potential of ultra-thin silver–copper film masks offer new possibilities for practical applications of multifunctional masks.

## 1. Introduction

The variability of the COVID-19 virus presents challenges for existing vaccines in achieving effective antiviral outcomes, posing a significant threat to human health [1]. Face masks act as a physical barrier, efficiently preventing mucosal droplets from entering the nasal and oral cavities. This minimizes the spread of respiratory droplets, a crucial measure in reducing the risk of COVID-19 and other respiratory infections [2,3]. However, currently available commercial face masks have certain limitations. While standard medical surgical masks effectively prevent bacteria or viruses from entering the respiratory system, they lack antibacterial or antiviral functionality. The widespread use of disposable filtering materials has resulted in the generation of a significant number of discarded face masks, not only increasing the potential risk of viral transmission but also imposing a new environmental burden [4]. This inadequacy becomes particularly prominent in the ongoing impact of respiratory diseases, such as COVID-19. Therefore, the imperative development of intelligent face masks with antibacterial and antiviral capabilities is crucial for public health [5,6,7].

Over the past few decades, it has been discovered that metal nanoparticles (NPs) possess potent antibacterial properties, with the potential to mitigate or eliminate the emergence of drug-resistant bacteria. Therefore, utilizing metal NPs for the development of functional face masks with antibacterial properties is of paramount importance for protection against infectious diseases [8,9,10,11,12]. In recent years, there has been significant progress in applying metal NPs to face masks with antibacterial capabilities. For instance, Kumar et al. utilized a dual-channel spray-assisted nanocoating mixture of chitosan and Cu NPs on a non-woven surgical face mask. The resulting face mask exhibits excellent photothermal properties, enabling rapid warming and the destruction of nanoscale viruses when exposed to sunlight [13]. Valdez-Salas et al. developed a novel nanomaterial by impregnating Ag NPs as a safety-enhancing factor, enhancing the antibacterial activity of surgical face mask fibers [14]. Despite the proven potent bactericidal effects of face masks containing metal NPs, effectively curbing the spread of respiratory diseases, the singular use of antibacterial and antiviral face masks presents certain limitations in promptly detecting and monitoring changes in health conditions.

The presence of gases such as nitrogen oxides (NO_x_) and volatile organic compounds in exhaled breath has been acknowledged as indicative biomarkers for various diseases and metabolic processes [15]. Changes in the concentration of NO_x_ molecules in exhaled breath have emerged as effective diagnostic indicators for respiratory conditions such as asthma, cystic fibrosis, and bronchiectasis [16,17]. The integration of real-time respiratory monitoring capabilities into face masks has become a pivotal direction in the intelligent development of face mask research. This involves the continuous surveillance of respiratory gas variations to detect and diagnose respiratory-related diseases [1,4,18]. For example, Mohammad et al. integrated an optical sensor into a thin face mask, creating an intelligent respiratory monitoring face mask. This face mask can distinguish COVID-19 infected individuals, recovered patients, and healthy individuals by detecting metabolic compounds in exhaled breath [19]. Zhang et al. have innovatively developed an intelligent face mask featuring a breathable and biodegradable self-powered respiratory sensor as its key component. This intelligent face mask employs a machine learning algorithm based on the bagged decision tree, achieving an impressive accuracy of 95.5% in distinguishing between the healthy group and three cohorts with chronic respiratory diseases, namely asthma, bronchitis, and chronic obstructive pulmonary disease [18]. Although intelligent face masks for health monitoring have achieved preliminary results, they typically integrate sensors to enable precise measurement of respiratory parameters. However, these intelligent face masks face challenges such as complex fabrication processes and higher costs, limiting their large-scale application.

In this study, we utilized the high-vacuum roll-to-roll (R2R) magnetron sputtering method to fabricate ultra-thin Ag–Cu films on non-woven fabrics for face masks, achieving multifunctionality. The ultra-thin Ag–Cu film face masks not only effectively deactivate *Escherichia coli* (*E. coli*) and *Staphylococcus aureus* (*S. aureus*) but also significantly reduce the titer of influenza A virus H1N1. Furthermore, the reaction of ultra-thin Ag–Cu films with NO_x_ exhibits a noticeable color change (from brownish-yellow to grey-white), which serves as an effective means for monitoring respiratory disease. This work demonstrates an innovative smart face mask with concurrent antibacterial, antiviral, and NO_x_-responsive functionalities. Its scalable manufacturability presents new opportunities for developing cost-effective, intelligent, and durable face masks.

## 2. Materials and Methods

### 2.1. Materials

Disposable medical surgical face masks and non-woven fabrics, with polyethylene terephthalate (PET) as the main component, were purchased from local markets. Sputtering experiments were performed using a 90 × 13 cm^2^ Ag–Cu target (Ag/Cu = 95:5 at%).

### 2.2. Fabrication of Ultra-Thin Ag–Cu Film

Before preparation, the non-woven fabric underwent a 30-s argon (Ar) plasma pretreatment to enhance adhesion between the fibers and the membrane layer. Subsequently, an R2R magnetron sputtering device was used to deposit the Ag–Cu film onto the non-woven fabric. The pretreated non-woven fabric was placed on an unwind roller in an open chamber and wound onto a rewind roller. All chamber doors were closed, and the vacuum level in the chamber was sequentially reduced to 2.0 × 10 Pa and 5.0 × 10^−4^ Pa using mechanical and molecular pumps. Then, argon gas with a purity of 99.999% was introduced into the chamber at a flow rate of 400 sccm, resulting in a pressure of 3.0 Pa. An Ag–Cu film was deposited using a 0.2 kW DC power supply at a deposition rate of 35 nm/min. Approximately 8.6 s of deposition produced a 5 nm thick Ag–Cu film. Upon completion of deposition, the non-woven fabric was awaited at the rewind roller in the rewind chamber. The intake valve of the rewind chamber was opened to restore normal pressure, and then the door of the rewind chamber was opened to retrieve the non-woven fabric [20,21,22].

### 2.3. Fabrication of Ultra-Thin Ag–Cu Film Face Mask

The non-woven fabric with deposited Ag–Cu film was used as the outermost layer of the mask’s three-layer structure, and ultra-thin Ag–Cu film face masks were manufactured using commercial surgical face mask production equipment. The specific preparation process is as follows: the inner layer of soft non-woven fabric, the middle layer of melt-blown fabric, and the outermost layer of ultra-thin Ag–Cu film non-woven fabric are stacked together to form the main body of the face mask through high-frequency welding. The welded face mask body is then folded using three adjustable inclined planes, gradually narrowing the incline to create a folding structure. Subsequently, the folding structure is compressed, while on one side of the non-woven fabric, metal wires are fed and sewn along the edge to attach ear loops. The edges are then compressed to complete the preparation of the ultra-thin Ag–Cu film face mask [23].

### 2.4. Characterization

SEM images of the ultra-thin Ag–Cu film non-woven fabric were taken by a field emission scanning electron microscope (Regulus 8230, Hitachi, Tokyo, Japan). The elemental distribution was analyzed using energy-dispersive X-ray spectroscopy (EDS, XFlash 6|100, Bruker, Karlsruhe, Germany). The surface elemental distribution of the films were obtained by X-ray photoelectron spectroscopy (XPS, Axis supra, Kratos, Manchester, UK) with monochromatic Al Kα radiation (hν = 1486.7 eV). The XPS spectra were calibrated based on the binding energy of C 1s = 284.8 eV.

### 2.5. Antibacterial and Antiviral Activity Tests

The antibacterial and antiviral activity tests were conducted in accordance with ASTM E 2149 [24] and ISO 18184:2019 (E) [25] standards, respectively. For more details, see Supplementary Notes S1 and S2.

### 2.6. Filtration Efficiency Test

According to the NIOSH 42 CFR Part 84 standard, the filtration efficiency of ultra-thin Ag–Cu film masks was determined [26]. During the filtration efficiency testing of the masks, to ensure accuracy, appropriate fixed fixtures were used to securely connect the face masks to the testing equipment. Filtration efficiency results were continuously recorded during the continuous loading of a NaCl aerosol, and multiple measurements were taken to obtain an average value to ensure accurate data collection. The concentration of NaCl particles used did not exceed 200 mg/m^3^, with a count median diameter of 75 ± 20 nm. The testing conditions included maintaining a temperature of 25 ± 5 °C and a relative humidity of 30 ± 10%. The masks were fixed on fixtures, and the test airflow corresponded to the required filtration velocity. Dry, oil-free compressed air entered the aerosol generator at a certain flow rate, generating aerosol with a specific particle distribution. The aerosol underwent screening via a cascade impactor to discharge particles with the required size distribution at the aerosol outlet. Subsequently, the aerosol was neutralized by electrostatic means, mixed evenly with filtered test air, and then entered the test area for testing through the mask. A photometer was used to measure the concentrations of NaCl aerosol in the upstream and downstream air, and the filtration efficiency of the tested samples was obtained by comparing the concentrations upstream and downstream.

### 2.7. Adhesion Fastness Test

We employed the tape test method to assess the adhesion of the ultra-thin Ag–Cu film on non-woven fabric. To ensure the accuracy of the test, we applied a weight of 200 g on the insulating tape during the testing process, ensuring full contact between the testing tape and the fibers as well as the interstices of the non-woven fabric, thus obtaining more accurate measurement results. The specific test steps are as follows: affix the testing tape to the surface of the ultra-thin Ag–Cu film non-woven fabric, then place a 200-g weight on the testing tape. After 30 s, remove the weight and peel off the testing tape. Subsequently, repeat the above test procedure using a new testing tape. After 100 tests, observe any color changes on the surface of the ultra-thin Ag–Cu film non-woven fabric to evaluate its adhesion.

### 2.8. Breathability Test

The breathability of the ultra-thin Ag–Cu films was evaluated using an acrylic breathability tester. Circular samples with a diameter of 75 mm were prepared using a cutting tool. The samples were placed between the sealing rings of the upper and lower cups. The upper cup was filled with 200 mL of deionized water, and a certain amount of air was introduced into the lower cup. The size and number of bubbles in the upper cup of the ultra-thin Ag–Cu film face masks and surgical face masks were compared for evaluation.

## 3. Results and Discussion

### 3.1. Antimicrobial Mechanism and Breath NO_x_ Response

**Figure 1:**
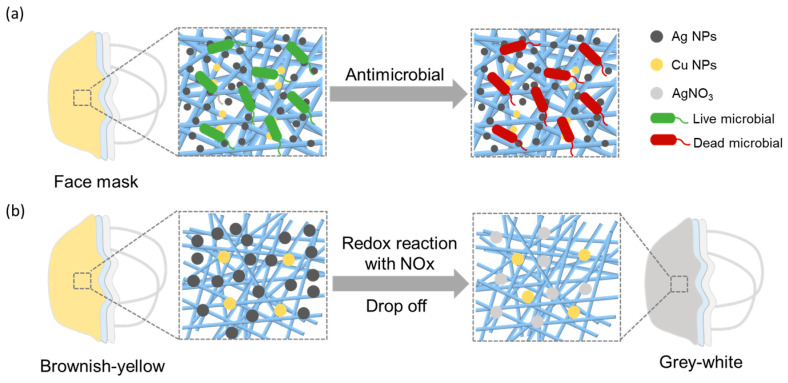
(**a**) The antimicrobial mechanism of Ag–Cu NPs. (**b**) The mechanism of Ag NPs response to NO_x_.

Ag–Cu NPs not only exhibit excellent antimicrobial effects but also demonstrate excellent responsiveness to oxidative gases such as NO_x_. As depicted in Figure 1a, Ag NPs attach to bacterial cell walls and membranes, followed by their penetration into the microbial interior. Inside, they disrupt intracellular structures such as mitochondria, vacuoles, and ribosomes, as well as biomolecules including proteins, lipids, and DNA. Additionally, they induce cell toxicity and oxidative stress through the generation of reactive oxygen species and free radicals, ultimately resulting in microbial inactivation [27]. Furthermore, Cu NPs can induce oxidative stress, leading to the disintegration of viral or bacterial membranes, interfering with viral activity, and enhancing the production of toxic compounds against microorganisms, ultimately achieving antimicrobial effects [28]. As shown in Figure 1b, under the action of Ag NPs, NO_x_ is oxidized to NO_2_^−^/NO_3_^−^, ultimately resulting in the formation of AgNO_3_ [29,30]. This reaction leads to the oxidation and detachment of Ag on the surface of the mask, ultimately causing a noticeable change in the mask’s color. By applying Ag–Cu NPs to masks, not only excellent antibacterial properties are imparted, but also respiratory monitoring functionality is achieved.

### 3.2. Preparation and Composition Analysis of Ultra-Thin Ag–Cu Film Face Mask

**Figure 2:**
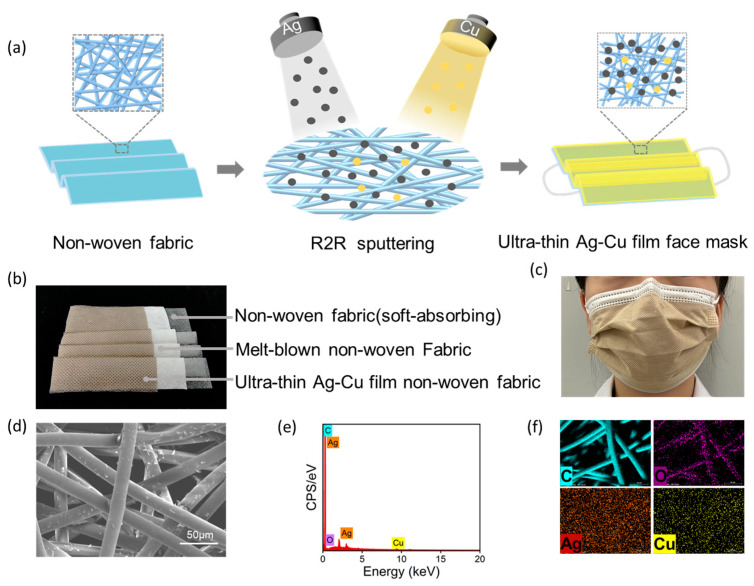
(**a**) Preparation process flowchart of ultra-thin Ag–Cu film face mask; (**b**) Optical photograph of the three-layer structure of the ultra-thin Ag–Cu film face mask; (**c**) Optical photograph of the ultra-thin Ag–Cu film face mask during actual wearing; (**d**) SEM images of the ultra-thin Ag–Cu film face masks; (**e**) Point-scanning EDS spectrum of the ultra-thin Ag–Cu film face masks; (**f**) Surface-scanning EDS spectroscopy of the ultra-thin Ag–Cu film face masks.

We functionalized the surface of non-woven fabric using the high-vacuum R2R magnetron sputtering method. As shown in Figure 2a, we deposited a 5 nm ultra-thin silver-copper film on the surface of the non-woven fabric. The prepared ultra-thin silver-copper film non-woven fabric was then used as the outer layer of the face mask to prepare the ultra-thin silver-copper film face mask. As illustrated in Figure 2b, the ultra-thin Ag–Cu film face masks are composed of an inner layer of soft non-woven fabric, an intermediate melt-blown fabric, and an outer layer of non-woven fabric with an ultra-thin Ag–Cu film. Optical and scanning electron microscopy (SEM) images of the ultra-thin Ag–Cu film face masks are presented in Figure 2c,d, respectively. From the SEM image, it can be observed that the average diameter of nonwoven fabric fibers is 18.4 μm, and the average pore size between fibers is 73.1 μm. The diameter of Ag–Cu NPs produced by magnetron sputtering is much smaller than the pores between the fibers. Therefore, Ag–Cu NPs can not only be uniformly distributed on the surface of nonwoven fabric fibers but also deposited into the pores of the surface fibers of the nonwoven fabric. As shown in Figure 2e, point-scanning EDS spectroscopy of the ultra-thin Ag–Cu film face masks indicates the presence of elements including C, O, Ag, and Cu on the surface of the ultra-thin Ag–Cu film face mask. In the surface scanning EDS spectrum of Figure 2f, the distribution of C and O elements aligns with the orientation of the fibers, indicating that C and O are the main components of the fibers, consistent with the primary composition of the PET non-woven fabric used. Additionally, Ag and Cu elements exhibit uniform distribution across the entire surface, further demonstrating that Ag and Cu nanoparticles can deposit not only on the surface fibers of the non-woven fabric but also in the interstices of the surface fibers.

### 3.3. Antimicrobial Performance of Ultra-Thin Ag–Cu Film Face Mask

The antibacterial performance of the ultra-thin Ag–Cu film face masks against *E. coli* and *S. aureus* was assessed using an oscillation method. Test samples included standard blank samples, standard control samples (cotton fabric), and the outer non-woven fabric of the ultra-thin Ag–Cu film face masks. (The blank sample was prepared using standard procedures on pure cotton fabric. This involved immersing the pure cotton fabric in a 15 g/L NaOH solution at 100 °C for 3 h, followed by multiple rinses with deionized water. Finally, the treated cotton fabric was bleached using a 3 g/L H_2_O_2_ solution, rinsed with water, and then dried). The antibacterial performance of the ultra-thin Ag–Cu film face masks was evaluated by assessing bacterial survival rates.

**Figure 3:**
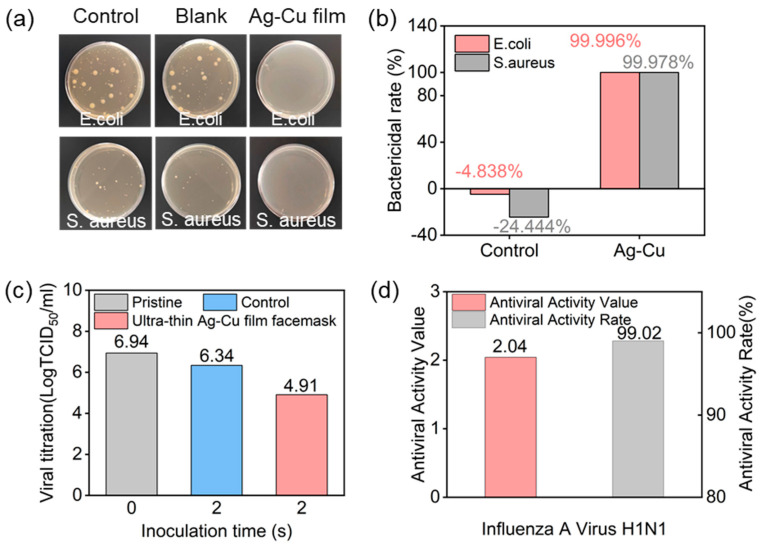
(**a**) Optical images of colony-forming unit plates for *E.coli* and *S. aureus* after 18 h of treatment using raw fabric, blank samples, and ultra-thin Ag–Cu film non-woven fabric; (**b**) Quantitative assessment of antibacterial properties based on *E. coli* and *S. aureus* survival rate; (**c**) The logarithmic values of the average virus titers for the control sample and ultra-thin Ag–Cu film face masks; (**d**) Antiviral activity values and antiviral activity rates of ultra-thin Ag–Cu film face masks against influenza A virus H1N1.

Figure 3a displays optical images of the three groups of samples after antibacterial testing, indicating minimal colony distribution on the surface of the ultra-thin Ag–Cu film. Figure 3b presents the antibacterial rates for the control samples and the ultra-thin Ag–Cu film face masks against *E. coli* and *S. aureus*. The antibacterial rates for the control samples against both bacteria were −4.838% and −24.444%, respectively, confirming that standard control samples could not inhibit bacterial growth. In contrast, the Ag–Cu film non-woven fabric exhibited antibacterial rates of 99.996% and 99.978% against *E. coli* and *S. aureus*, respectively, demonstrating excellent bactericidal efficacy for the ultra-thin Ag–Cu film non-woven fabric. These results indicate the outstanding antibacterial performance of the ultra-thin Ag–Cu film non-woven fabric against *E. coli* and *S. aureus*, as assessed through the described testing method. Additionally, we conducted tests on the antiviral performance of the ultra-thin Ag–Cu film face masks. Figure 3c,d illustrate the antiviral effects of the ultra-thin Ag–Cu film face masks against influenza A virus H1N1. Compared to the control sample with 0 contact, the average virus titer of the control sample after 2 h of incubation was 6.34, with an antiviral activity value of only 0.60. In contrast, the average virus titer for the ultra-thin Ag–Cu film face masks after 2 h of incubation was 4.91, with an antiviral activity value of 2.04 ± 0.14. This demonstrates the effective inactivation of influenza A virus H1N1 by the ultra-thin Ag–Cu film face mask, with an antiviral activity rate of up to 99.02%.

### 3.4. NO_x_ Response of Ultra-Thin Ag–Cu Film Face Mask

**Figure 4:**
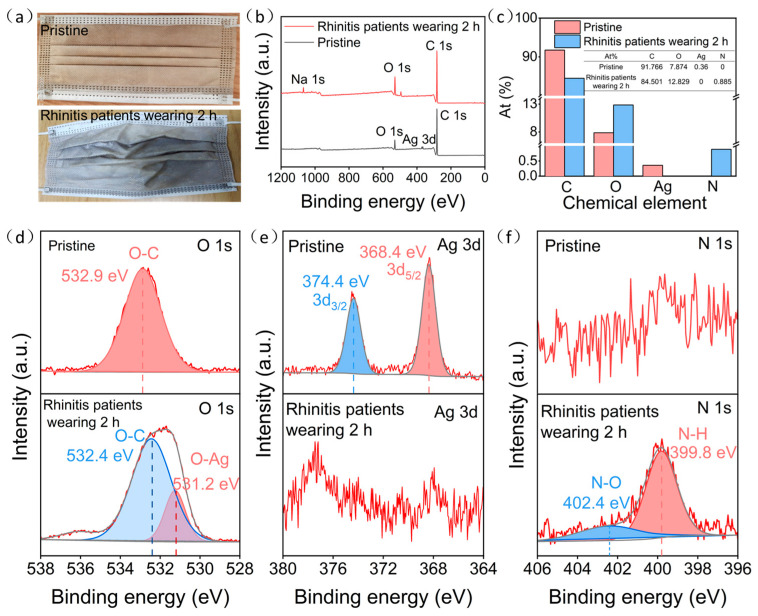
(**a**) The pristine ultra-thin Ag–Cu film face mask and the ultra-thin Ag–Cu film face mask worn by rhinitis patients for 2 h; (**b**) XPS survey spectra of the pristine ultra-thin Ag–Cu film face mask and the ultra-thin Ag–Cu film face mask worn by rhinitis patients for 2 h; (**c**) The atomic percentage of the pristine ultra-thin Ag–Cu film face mask and the ultra-thin Ag–Cu film face mask worn by rhinitis patients for 2 h; (**d**–**f**) Detailed spectra of the O 1s, Ag 3d and N 1s orbitals for the pristine ultra-thin Ag–Cu film face mask and the ultra-thin Ag–Cu film face mask worn by rhinitis patients for 2 h.

To assess the effectiveness of ultra-thin Ag–Cu film face masks in practical respiratory monitoring, we conducted a comparative analysis between the pristine ultra-thin Ag–Cu film masks and those worn by rhinitis patients for 2 h. As depicted in Figure 4a, the color transition of the ultra-thin Ag–Cu film face masks, shifting from their initial brownish-yellow tint to grey-white after being worn by rhinitis patients, suggests their potential utility in monitoring respiratory conditions. Examination of XPS spectra and elemental composition, as illustrated in Figure 4b,c, unveiled notable alterations. After 2 h of wear by rhinitis patients, the distinctive Ag 3d peak disappeared, concomitant with a reduction in elemental Ag composition from 0.36 to 0. This indicates the oxidation of surface Ag to AgNO_3_ and subsequent detachment. Furthermore, the appearance of the N 1s peak and an increase in elemental N composition from 0 to 0.885 further corroborate these findings.

To better elucidate the reaction process, we analyzed the spectra of O 1s, Ag 3d, and N 1s for both the pristine ultra-thin Ag–Cu film face masks and those worn by rhinitis patients. As shown in Figure 4d, the pristine ultra-thin Ag–Cu film face masks exhibited only one characteristic peak at 532.9 eV, corresponding to the O-C bond inherent to the non-woven fabric material. However, after 2 h of wear by rhinitis patients, additional characteristic peaks emerged at 532.4 eV for the O-C bond and at 531.2 eV for the O-Ag bond, indicating the presence of O-Ag bonds on the surface of the ultra-thin Ag–Cu film face masks [31]. As illustrated in Figure 4e,f, the pristine ultra-thin Ag–Cu film face masks displayed characteristic peaks of Ag 3d_5/2_ and Ag 3d_3/2_ at 368.4 and 374.4 eV, respectively, representing typical features of metallic Ag, confirming the presence of metallic Ag in the original masks [32]. However, after 2 h of wear by rhinitis patients, no characteristic peaks of Ag were observed. Instead, peaks appeared at 399.8 and 402.4 eV, corresponding to N-H and N-O bonds, respectively, consistent with the NO_2_^−^/NO_3_^−^ generated from the reaction of NO_x_ with Ag [33]. This further confirms that NO_x_ in the exhaled breath of rhinitis patients oxidized Ag in the ultra-thin Ag–Cu film face masks to AgNO_3_, ultimately leading to detachment from the mask surface.

### 3.5. Filtration Efficiency Breathability, and Durability of Ultra-Thin Ag–Cu Film Face Masks

**Figure 5:**
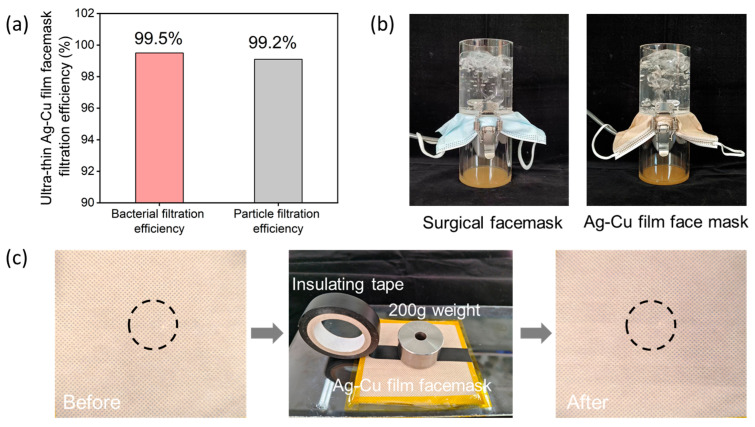
(**a**) Bacterial filtration efficiency and particle filtration efficiency of ultra-thin Ag–Cu film face masks. (**b**) Breathability testing of medical surgical face masks and ultra-thin Ag–Cu film face masks. (**c**) Adhesion testing of ultra-thin Ag–Cu film face masks.

In addition to antibacterial performance testing, we conducted filtration efficiency, breathability, and durability tests on the ultra-thin Ag–Cu film face mask. The filtration performance of face masks is one of their primary functions, as they effectively block airborne particles and particulate matter, thus safeguarding respiratory health. Therefore, the filtration efficiency of face masks is crucial in preventing the intrusion of bacteria, viruses, and other harmful substances. As shown in Figure 5a, we evaluated the filtration efficiency of ultra-thin Ag–Cu film face masks against *Staphylococcus aureus* and sodium chloride aerosols. The test results indicate a significant reduction in particulate matter concentration in the test air after passing through the ultra-thin Ag–Cu film face mask, with most *Staphylococcus aureus* and sodium chloride aerosols depositing on the face mask surface. This demonstrates the excellent filtration effectiveness of the ultra-thin Ag–Cu film face mask against *Staphylococcus aureus* and sodium chloride aerosols, with filtration efficiencies of 99.5% and 99.2%, respectively. Figure 5b demonstrates that the ultra-thin Ag–Cu film face mask maintains excellent breathability when compared to medical surgical face masks. Figure 5c presents photographs of the ultra-thin Ag–Cu film face mask after undergoing 100 rounds of adhesive tape testing, with circles indicating the test regions. It can be observed from the images that there is no noticeable change in the color of the face mask surface before and after testing, confirming the firm adhesion of Ag and Cu NPs to the surface of the non-woven fabric fibers. These tests collectively demonstrate the filtration effectiveness, breathability, and durability of the ultra-thin Ag–Cu film face mask, in addition to its antibacterial properties.

## 4. Conclusions

In summary, we achieved the scalable manufacturing of ultra-thin Ag–Cu film face masks through the R2R sputtering method. The 5 nm ultra-thin Ag–Cu film imparts outstanding antibacterial and antiviral properties to the face masks. The antibacterial rates against both *E. coli* and *S. aureus* were 99.996% and 99.978%, respectively. Moreover, they significantly reduced the titer of influenza A virus H1N1, with an antiviral activity rate of 99.02%. Additionally, the ultra-thin Ag–Cu film’s color-changing response to respiratory markers such as NO_x_ provides a simple monitoring method for early detection and changes in the condition of respiratory diseases. This opens up new possibilities for developing intelligent and durable face masks.

## Data Availability

Data are contained within the article.

## Supplementary Materials

The following supporting information can be downloaded at: https://www.mdpi.com/article/10.3390/ma17071574/s1, Supplementary Note S1: Antibacterial test; Supplementary Note S2: Antiviral activity test.
