# Supplementary material for: Sputtering-Deposited Ultra-Thin Ag–Cu Films on Non-Woven Fabrics for Face Masks with Antimicrobial Function and Breath NOx Response
Source: Materials (Basel). 2024 Mar 29;17(7):1574. doi: 10.3390/ma17071574 (PMC11012588; doi:10.3390/ma17071574)
Supplement: Supplementary file 1 [file materials-17-01574-s001.zip › materials-2921658-supplementary.pdf]

Supplementary Information

Sputtering-deposited ultra-thin Ag–Cu films on non-woven fabrics for face masks with antimicrobial function and breath NO_x response

Xuemei Huang,[1,2] Qiao Hu[1], Jia Li,[1,] ,Wenqing Yao[3], Chun Wang[4], Yun Feng[4] and Weijie Song,[1,2,*]

[1]Ningbo Institute of Materials Technology and Engineering, Chinese Academy of Sciences, Ningbo 315201, China

[2]University of Chinese Academy of Sciences, Beijing 100049, China

[3]Department of Chemistry, Tsinghua University, Beijing 100084, China

[4]Ningbo Customs Technology Center, Ningbo 315012, China

[*] Corresponding author: weijiesong@nimte.ac.cn

Supplementary Note S1. Antibacterial test.

In this study, the antibacterial activity of ultra-thin Ag–Cu film non-wovens was tested against *E. coli* and *S. aureus* using the oscillatory method. The control samples were untreated antibacterial non-wovens and the oscillatory flask method described in ASTM E 2149 was used with appropriate modifications. The test organisms, the Gram-negative bacterium *E. coli* (ATCC 29522) and the Gram-positive bacterium *S. aureus* (ATCC 6538), were obtained by incubating them in nutrient broth for 18 h at 37 °C and 130 rpm. They were then sub-cultured in nutrient broth and stored at 5 °C. Afterward, the samples were incubated at 37 °C and 130 rpm for 18 h. Untreated antibacterial fabric was tested as a control sample. The ultra-thin Ag–Cu film non-woven fabric samples, control samples, and standard blank samples were subjected to sterilization treatment at 103 kPa and 121 °C for 15 mins in the environment. Both *E. coli* and *S. aureus* were cultured in nutrient broth at 37 °C and 130 r/min for 18 h to prepare the inoculum. Then, a 10-fold serial dilution was performed using 0.03 mol/L PBS buffer, thoroughly mixed, and diluted until a viable cell count of 3×10^5 CFU/mL was obtained. This diluted inoculum was used for sample inoculation. A volume of 5 mL of the inoculated solution was separately placed into the vials containing the ultra-thin Ag–Cu film non-woven fabric samples, control samples, and standard blank samples. These three vials were then positioned on a reciprocating shaker under conditions of 24 °C and 250 r/min for 1 min for agitation, followed by subsequent sampling at "0" contact time. Post-sampling, the vials were subjected to cultivation under oscillation at 24 °C and 150 r/min in an oscillation incubator for 18 h. After the designated time, take 1 mL of the test solution from each flask and transfer it to a test tube containing 9 mL of 0.03 mol/L PBS buffer. Shake the test tube to mix the solution, and then perform a series of dilutions using a 10-fold dilution method. Repeat the above steps to dilute the test solution three times. Take 1 mL of the 3-fold diluted test solution

and pour it into a Petri dish. Add 15 mL of nutrient agar medium to the dish, allow it to solidify at room temperature, and invert the Petri dish. Incubate the dish at 37 °C for 48 h. (To reduce errors, perform three parallel tests on the same sample, and report the average value of the antibacterial rate.) After the designated time, manually count the colony-forming units (CFU) and calculate the average bacterial count. The concentration of viable bacteria in the sample flask can be calculated using the following formula:

$$W = Z \times N$$

Where W represents the concentration of viable bacteria in the sample flask. Z represents the bacterial count. N is the dilution factor, $N = 10^0, 10^1, 10^2, \ldots$

To evaluate the antibacterial performance of the ultra-thin Ag–Cu film non-woven fabric based on bacterial survival rate, the antibacterial rate can be calculated using the following formula:

$$Y = \frac{W_\mathrm{b} - W_\mathrm{c}}{W_\mathrm{b}} \times 100\%$$

Where Y represents the antibacterial rate. W_b represents the concentration of viable bacteria in the flask after 18 h of oscillation contact with the standard blank sample. W_c represents the concentration of viable bacteria in the flask after 18 h of oscillation contact with the antibacterial fabric sample or the untreated antibacterial fabric sample.

Supplementary Note S2. Antiviral activity test.

The antiviral activity testing method followed the ISO 18184:2029 standard. Control samples and ultra-thin Ag–Cu film non-woven fabrics were placed in sterile plates, and 200 μL of the virus was inoculated onto the control samples and ultra-thin Ag–Cu film non-woven fabric. After virus inoculation, 20 mL of SCDLP was immediately added to three control samples. After a 2 h contact, 20 mL of SCDLP broth was added to three control samples and three ultra-thin Ag–Cu film non-woven fabric samples to recover the remaining virus. The rinse solution was serially diluted into ten dilutions, and the viral infectivity titer was determined by the TCID50 method for the recovered virus. The antiviral activity is determined by the following equation:

$$M_v = Log10(V_a) - Log10(V_c)$$

Where, M_v is the antiviral activity value, Log10 (V_a) is the logarithmic average of three infectivity titer value immediately after inoculation of the control specimen, Log10 (V_c) is the logarithmic average of three infectivity titer value after specific contact time with the test specimen.
